# Does Early Mobilization Following Resection of Spinal Intra-Dural Pathology Increase the Risk of Cerebrospinal Fluid Leaks?—A Dual-Center Comparative Effectiveness Research

**DOI:** 10.3390/medicina60010171

**Published:** 2024-01-17

**Authors:** Michael Schwake, Sophia Krahwinkel, Marco Gallus, Stephanie Schipmann, Emanuele Maragno, Volker Neuschmelting, Moritz Perrech, Michael Müther, Moritz Lenschow

**Affiliations:** 1 Department of Neurosurgery, University Hospital Münster, 48149 Münster, Germany; sophia.krahwinkel@ukmuenster.de (S.K.); marco.gallus@ukmuenster.de (M.G.); stephanie.schipmann@googlemail.com (S.S.); emanuele.maragno@ukmuenster.de (E.M.); michael.muether@ukmuenster.de (M.M.); 2 Department of Neurological Surgery, University of California San Francisco, San Francisco, CA 94143, USA; 3 Department of Neurosurgery, University Hospital Bergen, 5009 Bergen, Norway; 4 Department of Neurosurgery, University Hospital Cologne, 50937 Cologne, Germany; volker.neuschmelting@uk-koeln.de (V.N.); moritz.perrech@uk-koeln.de (M.P.); moritz.lenschow@uk-koeln.de (M.L.)

**Keywords:** CSFL, intradural spinal tumors, mobilization, postoperative complications, spinal duraplasty

## Abstract

*Background and Objectives*: Prolonged bed rest after the resection of spinal intradural tumors is postulated to mitigate the development of cerebrospinal fluid leaks (CSFLs), which is one of the feared postoperative complications. Nonetheless, the empirical evidence supporting this conjecture remains limited and requires further investigation. The goal of the study was to investigate whether prolonged bed rest lowers the risk of CSFL after the resection of spinal intradural tumors. The primary outcome was the rate of CSFL in each cohort. *Materials and Methods*: To validate this hypothesis, we conducted a comparative effectiveness research (CER) study at two distinct academic neurosurgical centers, wherein diverse postoperative treatment protocols were employed. Specifically, one center adopted a prolonged bed rest regimen lasting for three days, while the other implemented early postoperative mobilization. For statistical analysis, case–control matching was performed. *Results*: Out of an overall 451 cases, we matched 101 patients from each center. We analyzed clinical records and images from each case. In the bed rest center, two patients developed a CSFL (*n* = 2, 1.98%) compared to four patients (*n* = 4, 3.96%) in the early mobilization center (*p* = 0.683). Accordingly, CSFL development was not associated with early mobilization (OR 2.041, 95% CI 0.365–11.403; *p* = 0.416). Univariate and multivariate analysis identified expansion duraplasty as an independent risk factor for CSFL (OR 60.33, 95% CI: 0.015–0.447; *p* < 0.001). *Conclusions*: In this CER, we demonstrate that early mobilization following the resection of spinal intradural tumors does not confer an increased risk of the development of CSFL.

## 1. Introduction

One of the common strategies used to prevent postoperative cerebrospinal fluid leakage (CSFL) after spinal intradural tumor resection involves implementing a regimen of postoperative bed rest. This decision is predicated upon the supposition that a horizontal position facilitates reduced intradural pressure, which, in turn, promotes the improved healing of the durotomy site and thereby minimizes the risk of CSFL development [1,2]. On the other hand, prolonged bed rest after surgery is associated with a number of medical complications, including nosocomial infections, thrombosis and pulmonary embolism, i.e., potentially fatal consequences of surgical interventions for frequently benign conditions [2,3]. In an attempt to challenge the traditional regimen of prolonged postoperative bed rest after intradural spine surgery, previous publications have shown mixed results [2,3,4,5,6]. Examples include a study by Radkliff et al. that found that early or late mobilization had no effect on CSLF after planned durotomies, while Lee et al. even found higher CSLF rates after prolonged bed rest [2,3].

Our recent investigation involved a monocentric cohort study that did not reveal that bed rest had a statistically significant effect on reducing the incidence of CSFL [7]. Instead, our analysis highlighted that the surgical approach itself played a considerably more substantial role in determining the risk of CSFL. Specifically, more invasive approaches, the presence of large voids and, notably, expansion duraplasty were associated with an increased risk of CSFL. Nevertheless, it is important to acknowledge the presence of possible confounding factors in the available retrospective studies, i.e., significantly more patients with pathologies in the caudal parts of the spine were ordered to undertake bed rest.

The presence of these co-founders and the multitude of possible or suspected risk factors and their potential interaction represent the main challenge in the prevention of CSFL. The precise identification or assessment of the significance of individual risk factors for postoperative CSFL fistula formation would constitute a significant advance in clinical patient care, both in terms of morbidity and mortality and in economic terms.

Ideally, this would require a prospective multicenter study, which is unlikely to be available in the foreseeable future due to the comparatively low frequency of procedures, even at large institutions. In this study, we present the first ever published comparative effectiveness research (CER) investigating the effects of postoperative mobilization in two comparable cohorts of patients who underwent a resection of spinal intradural tumors or vascular malformations. This comparison was enabled because the two academic neurosurgical institutions implicate different postoperative management strategies; in the first, patients were ordered to undertake a three-day period of bed rest, while in the second, patients were mobilized early after surgery. The value of such comparative studies was recently underlined in clinical neuro-oncology. A study compared different treatment approaches in two Norwegian hospitals to evaluate the treatment of low-grade gliomas, leading to a change in the standard treatment paradigm for this entity [8].

The primary outcome of this study was the rate of CSFL in each cohort; furthermore, we investigated the further potential risks of prolonged rest and the risk factors for the development of CSFL after the resection of spinal intradural pathologies.

## 2. Materials and Methods

### 2.1. Study Design

For this retrospective comparative effectiveness research, we included all patients who were operated on for spinal intradural tumors and vascular malformations between 2012 and 2020. Patients were excluded from the study if they had missing or incomplete records or if they were under the age of 18 at the time of the operation.

The primary aim of the study was to investigate the possible differences in the rate of postoperative CSFL observed between the two bed rest regimes. CSFL was defined as leakage through the operative wound or the development of a pseudomeningocele necessitating operative or non-operative treatment.

Postoperative bed rest was ordered according to the institutional standard of care: In center 1, patients were mobilized as soon as possible after surgery, whereas in center 2, patients were mobilized following bed rest for three days. Patients were excluded in cases that deviated from the center’s standard of bed rest ordered by the treating surgeon due to case-specific factors, as well as non-compliance on the part of the patient. Dural closure was performed at the discretion of the attending surgeon.

The following potential influencing variables were examined: age, sex, type of pathology, location of pathology in the spine, preoperative and postoperative neurologic status using the McCormick scale [9], surgical approach (laminectomy, hemilaminectomy, dorso-lateral approach, laminoplasty), primary procedure or recurrence, duration of surgery, type of duraplasty (suture, expansion duraplasty, use of adhesives), length of hospital stay, and perioperative complications. The location of the pathology in the spine was further subdivided into those that were cranial and caudal to the sixth thoracic vertebra (T-6).

To compare the two cohorts, case matching was performed, and a case–control analysis was conducted, in accordance with the Strengthening the Reporting of Observational Studies in Epidemiology (STROBE) guidelines for case–control studies [10]. The study was executed in accordance with the Declaration of Helsinki. Ethical approval was obtained from the institutional review board (reference number 2021-714-f-S; approval date: 15 February 2022). Patient consent was not required by the institutional review board due to the retrospective, non-interventional study design.

### 2.2. Surgical Procedure

In both centers, analogous surgical approaches were employed: The patients were operated on in prone position, and after positioning, the location of the pathology was determined using fluoroscopy. Intraoperative neurophysiological monitoring was utilized in all procedures. For the resection of juxtamedullary pathologies, unilateral laminotomy was the preferred surgical approach. On the other hand, laminoplasty was the preferred approach for intramedullary tumors; in this case, the lamina was reinserted and fixed using plates and screws. Laminectomy and the dorso-lateral approach were chosen according to the case-specific factors.

The standard dural closure consisted of a watertight suture and an onlay seal (generally using TachoSil^®^; Takeda GmbH, Berlin, Germany); in the case that the cord swelling of a large defect in the dura occurred, duraplasty was performed. Duraplasty was performed using a xenogeneic dura substitute that was sutured to the dura in a watertight fashion. A watertight dural closure was confirmed intraoperatively using the Valsalva maneuver prior to subsequent wound closure. Other approaches and types of duraplasty were used on a case-by-case basis at the discretion of the treating surgeon. The resection of meningiomas was performed according to Simpson grade 2, which was achieved via the coagulation of the dural attachments in all cases described in this manuscript.

### 2.3. Statistics

Statistical analysis was performed using the software IBM SPSS Statistics 24.0 (IBM Corp., New York, NY, USA). To reduce confounding factors, we performed a semi-accurate matching, taking into account the following parameters: the dura closure technique, intramedullary and extramedullary tumors, the location in the spine cranial and caudal to T-6, as well as the surgical approach (laminectomy in comparison to non-laminectomy).

Categorical variables are shown as absolute and relative frequencies. Parametric values are presented in mean and standard deviation (SD). Non-parametric values are presented as the median and interquartile range (IQR). Two-tailed Student *t*-tests were used as the parametric tests and two-sided Mann–Whitney U-tests (MWU) were used as the non-parametric tests. Fisher’s exact test was performed to compare groups of binary categorical variables and the Chi-square test was used in case of multiple variables. Missing data were eventually excluded in the single calculation.

The odds ratio (OR) for the development of CSFL in both cohorts, including 95% confidence intervals (CI), was calculated. Multivariate analyses were performed using binary logistic regression. A probability value less than *p* < 0.05 was considered statistically significant.

## 3. Results

In the retrospective analysis, we included 451 cases with spinal intradural pathologies operated on in both centers between 2012 and 2020. In total, 312 (69.8%) of the cases were operated on in the bed rest center while 136 (30.16%) were operated on in the early mobilization center. In the bed rest center, CSFL was observed in three cases (0.96%); this is compared to four cases (2.94%) in the early mobilization center, showing no significant difference between the two groups (*p* = 0.117).

To facilitate a more robust comparison between the centers, we conducted a case–control matching accordingly and continued the analysis with 101 patients from each center, as described above.

The median age of patients treated at center 1 was significantly lower (40.5 years) than that of the patients treated at center 2 (57.9 years). The gender distribution was not significantly different between the two centers (49% and 44% female patients). In both groups, most intradural lesions were located juxtamedullary (74.3% and 81.2%, respectively) and below the 6th thoracic vertebra (83.2% and 74.4%, respectively). The duration of the surgical procedure did not differ significantly between the centers (mean was 240 and 228 min). The surgical indications differed significantly between the two groups: The most frequent indications in center 1 were the resection of neurinomas (28.7%), meningiomas (13.9%), and juxtamedullary ependymomas (15.8%). In contrast, the most prevalent surgical indications in center 2 were the resection of meningiomas (37.6%), neurinomas (22.8%) and others (12.9%). The preoperative neurological status, as assessed by the McCormick scale, differed significantly between the two centers, with a higher number of patients with no or mild deficit In center 2. The intraoperative details did not differ significantly between the two centers: the surgical approach was mostly performed by avoiding laminectomy, which was performed in only about 10% of cases in both centers, and dural closure was performed in more than 90% of cases in both centers via direct dural suture with or without an additional sealant.

The general patient characteristics are detailed in Table 1 and the intraoperative details are detailed in Table 2.

In the matched analysis, the occurrence of postoperative CSFL was not significantly different between the patients in the bed rest cohort (2/101, 1.98%) and the early mobilization cohort (4/101, 3.96%; *p* = 0.683); thus, we found no association between early mobilization and CSFL (OR 2.041, 95% CI: 0.365–11.403; *p* = 0.416).

On the other hand, we could not observe an association between postoperative bed rest and the occurrence of potential complications, such as deep venous thrombosis, pulmonary embolism, wound infection, or other hospital-acquired infections (all *p* > 0.05), although the rate of wound infections was slightly higher in the bed rest cohort 6 (5.9% in comparison to 1 (1%), *p* = 0.119).

There was no association with a longer length of hospital stay (LOS). However, we did notice a tendency for a lower LOS in the early mobilization cohort (7.39 days, ±6.31) in comparison with the bed rest cohort (8.20 days, ±7.21), but this was not statistically significant (*p* = 0.399, Table 3).

In the subsequent univariate analysis, we found that the risk of CSFL was increased in the case of expansion duraplasty, as 3 out of 18 (16.67%) patients with a duraplasty developed a postoperative CSFL, compared to 3 out of 184 (1.63%) with direct suture (*p* = 0.010). Other possible risk factors, including sex, the intra- vs. juxtamedullary location, the surgical approach, primary or revision surgery, prolonged bed rest or the location within the spine in relation to T-6, were not associated with higher rates of CSFL (Table 4). This was confirmed by a multivariate binary logistic regression analysis that encompassed the surgical approach, recurrent surgery, bed rest, and expansion duraplasty, revealing that expansion duraplasty is an independent risk factor for CSFL (OR 60.33, 95% CI: 0.015–0.447; *p* < 0.001).

## 4. Discussion

Postoperative CSFL after the resection of spinal intradural tumors is one of the feared complications in spine surgery, given its potential to precipitate additional complications including meningitis and revision surgeries. Such outcomes are often associated with a prolonged LOS, escalated treatment expenses, and adversely impacted patient outcomes [1,7,11,12]. To mitigate the risk of CSFL, extended postoperative bed rest is commonly employed, as it is believed to foster durotomy closure by reducing intradural hydrostatic pressure [2,4,13].

However, in this comparative dual-center effectiveness research (CER) study, we were unable to substantiate any advantage of prolonged bed rest after the resection of intradural tumors to prevent CSFL.

Although the questionable benefit of postoperative bed rest has been demonstrated in a number of retrospective and prospective studies for unintentional durotomies, data on optimal postoperative management after planned durotomies are sparse and mainly based on monocentric retrospective studies with limited patient numbers. Accordingly, despite the relative frequency of these procedures in everyday neurosurgery, clinical management varies widely and is often based on institutional guidelines or individual surgeon preference, as evidenced by the opposing bed rest regimens of the two university hospitals compared in our study. Compared with classical retrospective studies, randomized controlled trials are generally considered to provide the highest-quality evidence, but randomization is not feasible in the case of postoperative bed rest and these studies are frequently limited in their applicability to routine care because of strict inclusion criteria. In contrast, a CER analysis with a matched patient cohort from two large university hospitals allows a direct comparison of treatment alternatives based on patient-relevant outcome parameters and under less standardized everyday conditions, thus representing a compromise between the aforementioned study designs.

In this context, the value of CER was recently underlined by a study in clinical neuro-oncology. The study compared different treatment approaches in two Norwegian hospitals in order to evaluate the treatment of low-grade gliomas, leading to a change in the standard treatment paradigm for this entity [8]. Accordingly, we believe that the CRE analysis presented in this paper provides the highest-quality data available to date to support clinical decision making regarding postoperative bed rest after planned spinal durotomy; this reaffirms earlier findings showing that postoperative bed rest does not significantly contribute to the development of CSFL. Furthermore, our findings suggest that other risk factors may hold greater significance in the occurrence of CSFL [2,5,7,14].

Several studies have shown that the early mobilization of patients after surgery has several advantages, including the prevention of nosocomial infections, deep vein thrombosis and pulmonary embolism. In addition, adopting a non-prolonged bed rest approach may offer the potential to expedite the recovery process, resulting in a shortened length of hospital stay (LOS) and reduced treatment costs [2,3,4,5,6,15]. Interestingly, in this study, we did not find any potential disadvantages of a prolonged bed rest for three days after surgery. In both cohorts, the potential complications of bed rest were very similar, with equal rates of thromboembolic cases and nosocomial infections. Moreover, in both cohorts, the LOS was not significantly different. One potential reason could be that both centers base their discharge policies on other factors. However, the LOS is assuming growing importance, particularly considering escalating financial considerations in contemporary healthcare practices and that hospitals are likely to place greater emphasis on shortening the LOS.

**1.**  **Localization of tumors**

Taking into consideration the intradural pressure hypothesis, one would expect a higher incidence of CSFL following intradural surgery in the caudal parts of the spine. Consequently, we matched patients based on pathologies that were cranial and caudal to T-6; this served as the pivotal point, based on previous findings identifying a hydrostatic independence point (independence of csf pressure from body position) between C-6 and T-5 [16]. Notably, our results revealed equivalent rates of postoperative CSFL. Furthermore, only one patient in each cohort developed CSFL following lumbar intradural surgery, thus confirming previous results not showing higher rates of CSFL in the lumbar spine [2,4,10,11,14,17,18].

**2.**  **Dura closure**

While primary suture is the mainstay for dural closure following planned durotomy, most surgeons add further (liquid or patch) sealants, either alone or as adjuncts, to ensure watertight closure. However, the benefit of these additional means is uncertain.

In this study, a primary dural suture with a patch sealing was used in the majority of cases, and a CSFL developed in 2% of cases, resulting in a slightly lower overall incidence than previously reported in the literature following this closure technique. Here, the average risk of postoperative fistula development following planned spinal dural incisions is reported within the means of 0 to 10% [11,15,19].

We were able to confirm one independent risk factor for CSFL. Expansion duraplasty led to a significantly higher rate of CSFL, as previously described [7], while our findings support that direct dural suture, augmented with a sealant patch, offers the best results regarding CSFL prevention [14,20,21,22,23].

Accordingly, we recommend performing direct suturing whenever possible. In addition, augmentation using additional sealants is recommended [7,20]. In case of expansion duraplasty, we recommend using xenogeneic bovine pericardium, as these have offered the best results in the past [24]. However, although unavoidable in many cases, the decision to perform expansion duraplasty must be weighed against the increased risk of CSFL, and patients must be informed accordingly preoperatively.

**3.**  **Laminectomy versus hemilaminectomy**

In comparison to other studies [7,25,26,27,28], we could not confirm that bilateral laminectomy is a risk factor for the development of CSFL after the resection of intradural tumors. However, in both cohorts, full laminectomy was performed very rarely, thus restricting the power of these results. Laminectomy may favor the development of CSFL as the absence of an abutment or barrier between the dura, muscle, and soft tissue creates a large gap, which has been hypothesized to favor the development of CSFL. In contrast, minimal invasive approaches, e.g., uni-lateral hemilaminectomy, create very small tissue voids, with the surrounding tissue serving as an abutment and enabling the better sealing of the durotomy site. This is comparable with the case of laminoplasty, where the reinserted laminae protect the durotomy site and prevent CSFL [7].

Accordingly, in terms of the implications for clinical practice, we therefore recommend minimal invasive approaches when possible. When a bilateral approach is preferred, we suggest performing a laminoplasty. In addition to the potential risk of CSFL, laminectomy has further disadvantages affecting patient outcomes, such as back pain and the possibility of post-laminectomy kyphosis. However, if it is unavoidable, we recommend void filling with autologous fat tissue, as previously described [7,25,29,30,31].

**4.**  **Limitation**

The primary limitation of this study lies in its retrospective design, coupled with the relatively limited number of patients enrolled. Nevertheless, given the low prevalence of spinal intradural tumors, executing a prospective randomized controlled trial would prove considerably challenging. As a viable alternative in the future, a large-scale registry study could be considered. A further limitation is the different age distribution in both groups, with significantly younger patients being treated in the bed rest center than in the early mobilization center. Nonetheless, the mean age in both groups—40.47 and 57.89 years, respectively—does not appear to indicate clinically relevant differences. In addition, patients in the early mobilization group had a significantly worse preoperative neurological status, as shown by differences in the McCormick scale, but we do not believe that this affects the results. Similarly, we do not consider the differences in the treated entities in the two centers to be a limitation that is relevant to the conclusions of this study, given that Simpson grade 2 resection was performed in all cases and thus dural closure was not affected. In this regard, another potential bias lies in differences in the surgical practice and expertise in the treatment of intradural spinal pathologies at the two centers. However, all cases were treated by experienced spine surgeons at large academic institutions with high caseloads of intradural spinal pathologies, without significant differences in terms of the surgical approach or dural closure technique used, suggesting no relevant differences in the quality of care.

## 5. Conclusions

The early mobilization of patients after intradural spinal procedures does not increase the risk of CSFL and should be considered as a standard treatment to avoid perioperative complications based on our results. Minimally invasive procedures in combination with a watertight primary dural closure and an additional dural sealant appear to be a decisive factor in the prevention of CSFL, while expansion duraplasty should be omitted if possible. Further investigations are still required to verify these results.

## Figures and Tables

**Table 1:** Patient characteristics, SD: Standard deviation.

	Center 1 (No Bed Rest) *n* = 101	Center 2 (Bed Rest) *n* = 101	*p* Value
Female sex (N, %)	50 (49.0%)	44 (43.6%)	0.481
Age (Mean, ±SD)	40.47 (±12.6) years	57.89 (±15.6) years	<0.001
Relationship to the spinal cord			0.310
Intramedullary (N, %)	26 (25.7%)	19 (18.8%)	
Juxtamedullary (N, %)	75 (74.3%)	82 (81.2%)	
Level in the spine (N, %)			0.169
Cranial to T-6 (N, %)	17 (16.8%)	26 (25.7%)	
Caudal to T-6 (N, %)	84 (83.2%)	75 (74.4%)	
Spinal region			0.207
Cervical spine (N, %)	4 (36.4%)	7 (63.6%)	
Thoracic spine (N, %)	41 (45.1%)	50 (54.9%)	
Lumbar spine (N, %)	56 (56.0%)	44 (44.0%)	
Surgical indication			0.002
Arteriovenous fistula (N, %)	1 (0.99%)	4 (3.96%)	
Intramedullary ependymoma (N, %)	12 (11.88%)	9 (8.91%)	
Juxtamedullary ependymoma (N, %)	16 (15.84%)	3 (2.97)	
Hemangioblastoma (N, %)	6 (5.94%)	3 (2.97)	
Meningioma (N, %)	14 (13.86%)	38 (37.62%)	
Metastasis (N, %)	6 (5.94%)	4 (3.96%)	
Nerve sheath tumor (N, %)	5 (4.95%))	4 (3.96%)	
Neurinoma (N, %)	29 (28.71%)	23 (22.77%)	
Other (N, %)	11 (10.89%)	13 (12.87%)	
Preoperative neurological status (McCormick scale) (N, %)			0.006
1	54 (53.6%)	57 (56.4%)	
2	18 (17.8%)	33 (32.7%)	
3	16 (15.8%)	6 (5.9%)	
4	10 (9.9%)	2 (2.0%)	
5	3 (3.0%)	3 (3.0%)	

Values are presented as mean ± SD or number of patients (% per column).

**Table 2:** Intraoperative details.

	Center 1 (No Bed Rest) *n* = 101	Center 2 (Bed Rest) *n* = 101	*p* Value
Duration of surgery (Mean, ±SD)	240.77 (±104.9) min.	228.32 (±107.6) min.	0.426
Surgical approach			>0.999
Laminectomy (N, %)	9 (9.9%)	9 (9.9%)	
Non laminectomy (N, %)	92 (91.1%)	92 (91.1%)	
Dura closure			0.806
Direct suture ± sealant (N, %)	93 (92.1%)	91 (90.1%)	
Expansion duraplasty (N, %)	8 (7.9%)	10 (9.9%)	
Primary surgery			0.481
Yes (N, %)	89 (88.1%)	92 (91.1%)	
No (N, %)	12 (11.9%)	8 (7.9%)	

Values are presented as mean ± SD or number of patients (% per column).

**Table 3:** Postoperative patient data.

	Center 1 (No Bed Rest) *n* = 101	Center 2 (Bed Rest) *n* = 101	*p* Value
Postoperative neurological status (McCormick scale) (N, %)			0.014
1	55 (54.5%)	63 (62.4%)	
2	17 (16.8%)	28 (27.7%)	
3	16 (15.8%)	6 (5.9%)	
4	10 (9.9%)	3 (3.0%)	
5	3 (3.0%)	1 (1.0%)	
Bed rest (N, %)	0 (0%)	101 (100%)	<0.001
Cerebrospinal fluid leakage			0.683
Yes (N, %)	4 (4.0%)	2 (2%)	
No (N, %)	97 (96%)	99 (98%)	
Length of hospital stay (Mean, ±SD)	7.39 (±6.3) days	8.20 (±7.3) days	0.399
Complications			
Wound infection (N, %)	1 (1.0%)	6 (5.9%)	0.119
Urinary tract infection (N, %)	1 (1.0%)	0	>0.999
Deep venous thrombosis (N, %)	1 (1.0%)	0	>0.999
Other (N, %)	7 (6.9%)	5 (5.0%)	>0.999

Values are presented as mean ± SD or number of patients (% per column).

**Table 4:** Comparison of patients with and without cerebrospinal fluid leakage.

	With CSFL (*n* = 6)	Without CSFL (*n* = 196)	*p* Value
Sex			>0.999
Male (N, %)	3 (3.2%)	91 (96.8%)	
Female (N, %)	3 (2.8%)	105 (97.2%)	
Localization			>0.999
Intramedullary (N, %)	1 (2.2%)	44 (97.8%)	
Juxtamedullary (N, %)	5 (3.2%)	152 (96.8%)	
Surgical approach			0.091
Laminectomy (N, %)	2 (11.1%)	16 (88.9%)	
Non-laminectomy (N, %)	4 (2.2%)	180 (97.8%)	
Primary surgery			0.110
Yes (N, %)	4 (2.2%)	178 97.8%)	
No (N, %)	2 (10.0%)	18 (90.0%)	
Dura closure			0.010
Direct suture ± sealant (N, %)	3 (1.6%)	181 (98.4%)	
Expansion duraplasty (N, %)	3 (16.7%)	15 (83.3%)	
Bed rest			0.683
Yes (N, %)	2 (2.0%)	99 (98.0%)	
No (N, %)	4 (4.0%)	97 (96.0%)	
Level in the spine			>0.999
Cranial to T-6	1 (2.3%)	42 (97.7%)	
Caudal to T-6	5 (3.1%)	154 (96.9%)	

Values are presented as mean ± SD or number of patients (% per row).

## Data Availability

The datasets generated and/or analyzed in this study are available upon reasonable request.
